# Selecting sorting centres to avoid long distance transport of weaned beef calves

**DOI:** 10.1038/s41598-020-79844-4

**Published:** 2021-01-14

**Authors:** T. Morel-Journel, E. Vergu, J.-B. Mercier, N. Bareille, P. Ezanno

**Affiliations:** 1 INRAE, Oniris, BIOEPAR, 44300 Nantes, France; 2 INRAE, MaIAGE, Université Paris-Saclay, 78350 Jouy-en-Josas, France; 3 Terrena Innovation, La Noëlle, 44155 Ancenis, France

**Keywords:** Scientific data, Zoology, Computational biology and bioinformatics

## Abstract

The transport of weaned calves from cow–calf producers to fatteners is a general concern for the young bull industry due to its documented negative impact on the welfare, health and performance of the animals. These transfers are often managed by intermediaries who transport weaned calves to sorting centres, where they are grouped into batches before being sent to fattening units. In this study, we present an algorithm to limiting these transfer distances by appropriately selecting the sorting centre through which they must go. We tested the effectiveness of this algorithm on historical data from a French beef producer organization managing 136,892 transfers using 13 sorting centres. The results show a decrease in the transfer distances compared to the historical record, especially for the calves travelling over long distances (− 76 km, i.e. 18% on average for the 33% longest transfers). Moreover, the distribution of calves between the sorting centres proposed by the algorithm reveals differences in their efficiency in minimizing transfer distances. In addition to its usefulness as a management tool for the daily transport of cattle, this algorithm provides prospects for improving the management of the sorting centres themselves.

## Introduction

Animal handling and transportation is a general concern for the livestock industry for a variety of farm animals^1–3^. In addition to the risks of disease transmission inherent in any commercial movement of animals between holdings^4^, transport itself also raises issues concerning the welfare and health of livestock. Indeed, these transfers can generate stress in livestock, both physiological or psychological^5–7^, with more detrimental effects on the animals as the transport distance increases^8–10^. These concerns are paramount for the young bull industry, because an important proportion of weaned beef calves are transported between farms during their lives.

We take the example of the French young bull industry for this study, which is structured around two types of actors involved in the transfer of weaned beef calves^11^. On the one hand, cow–calf producers rear suckling beef calves with the dam until the age of seven to nine months. On the other hand, fatteners buy weaned calves from cow–calf producers and take care of their fattening until they are slaughtered. The objective of the latter is to increase the bodyweight of the animals purchased in order to maximise their resale value. Those two types of actors can be located in separated areas. For instance, cow–calf producers are usually in pasture-based systems in rural areas of Central France, while fatteners are located in regions with intensive crop production, such as North-western France. Since these two facets of the beef cattle industry are managed in different locations, calves are moved, potentially over long distances. In order to match supply and demand and to facilitate these transfers, third party intermediaries may group weaned calves from potentially several cow–calf producers into batches, and sell them at once to fatteners. While some of these intermediaries are independent, about half of French beef production involves cooperatives which manage the creation and transport of batches using sorting centres^12^. Thus, weaned calves are usually first transported from the cow–calf producer to a sorting centre, and then possibly through other sorting centres, until the batch to which they are assigned to is finally sent to a fattening farm.

These multiple transfers can amount to large total distances travelled by the weaned calves, with expected deleterious effects on livestock. Firstly, transport distance is negatively correlated with the performance of young bulls^8,13^. Notably, Herve et al.^13^ estimated an average decrease of average daily gain (ADG) during fattening of − 11 g/day per 120 km over fattening periods of 313.4 days on average. Secondly, transport is detrimental to the welfare and health of weaned calves^14^. Indeed, bovine respiratory disease (BRD) are disproportionally developed by calves during the first week after their arrival in fattening units, decreasing their survival rate and requiring a heavy use of antibiotics^10,15,16^. Transport distance has been identified as a factor increasing the incidence of BRD during this critical period^8,14,17^, which also has a negative impact on the performance of animals during fattening^18,19^. Reducing transport distances of weaned calves before fattening could therefore have direct and indirect repercussion on their survival and performance.

In this study, we aimed to minimize the total distance travelled by weaned beef calves during their transfer from cow–calf producer to fattener. To this end, we developed an algorithm for assigning calves to sorting centres based on the total distance travelled. The algorithm was constrained by the following two restrictions: (1) not to change the composition of the batches or their destination, and (2) always to respect the maximum capacity of each centre, i.e. the maximum number of calves they can host at the same time. This algorithm was tested on a dataset of 136,892 weaned calf transfers managed by *Terrena Production Bovine*, a French organisation of beef producers that operates 13 different sorting centres between 2010 and 2018. We used the algorithm to reassign every calf to one of the cooperative’s sorting centre, while respecting the original composition of the batches and the maximum capacity of the sorting centres. To evaluate the effectiveness of the algorithm, we compared the historical transfer distances according to the original dataset and the optimised transfer distances that would have been travelled with the new assignment by the algorithm.

## Results

### The algorithm reduces the longest transfer distances

**Figure 1:**
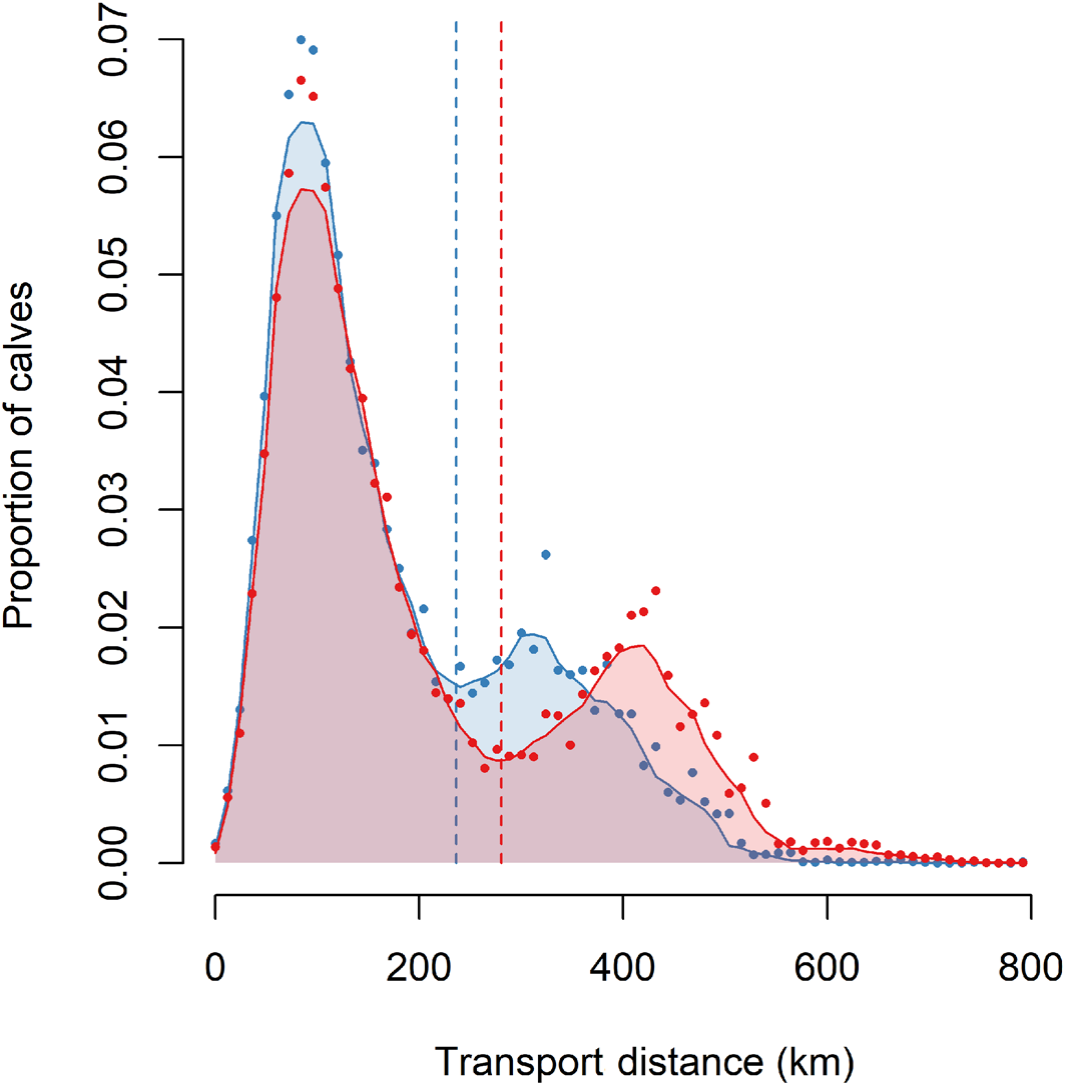
Distribution of the historical (red) and optimised (blue) transport distances. The dots represent frequencies of discrete classes of same size and the curves are obtained by smoothing of these frequencies (running median) The dashed vertical lines correspond to the two third of their respective distributions, used to separate short from long transfers.

We computed the distance travelled by each weaned calf according to the itineraries defined by the algorithm (optimised distances), and compared them with the distances computed according to the itineraries indicated in the *Terrena Production Bovine* dataset (historical distances). The distribution of these transfer distances are shown in Fig. 1. The distributions of the optimised and historical distances were clearly bimodal, with a “short-transfer” peak around 90 km for both distributions and a “long-transfer” peak around 420 km for the historical distances, and 310 km for the optimised distances. We considered a cut-off between the two peaks that was at the second third for both distributions. We have defined short and long transfers as those respectively below and above this threshold (at 294 km for historical distances and 245 km for optimised distances). The algorithm had a small impact on short transfers, which were reduced by − 8.6 km, i.e. 7.6% less than the historical ones. However, it had a stronger impact on the long transfers, which were reduced by 76 km on average, i.e. 18% less than the historical ones. The contribution of each centre to these long historical transfers was very heterogeneous (Table 1). Indeed, three sorting centres (numbered 9, 10 and 12) gathered a disproportionately large proportion of them, relative to their share of the overall transfers. Altogether, they accounted for 31% of all transfers, but 71% of long transfers.

**Table 1:** Distribution of the transfers of weaned calves depending on the first centre they went through according to the database.

Centre	Prop. of all transfers	Proportion of long transfers relative to:	
		All long transfers	The transfers through the centre
1	0.001	0.002	0.566
2	0.003	< 0.001	0.009
3	0.003	< 0.001	0.032
4	0.004	0.000	0.000
5	0.014	0.002	0.049
6	0.017	0.006	0.113
7	0.020	0.021	0.356
8	0.033	0.006	0.068
9	0.048	0.065	0.455
10	0.080	0.101	0.422
11	0.148	0.034	0.077
12	0.182	0.540	0.993
13	0.446	0.222	0.167

First column: the sorting centre considered. Second column: the proportion of all the transfers that went through a given centre. Third column, the proportion of long transfers (33% longest) that went through a given centre. Fourth column, the proportion of transfers through a given centre that were long-distance transfers (in the 33% longest).

### Transport distances relative to their minimum

**Figure 2:**
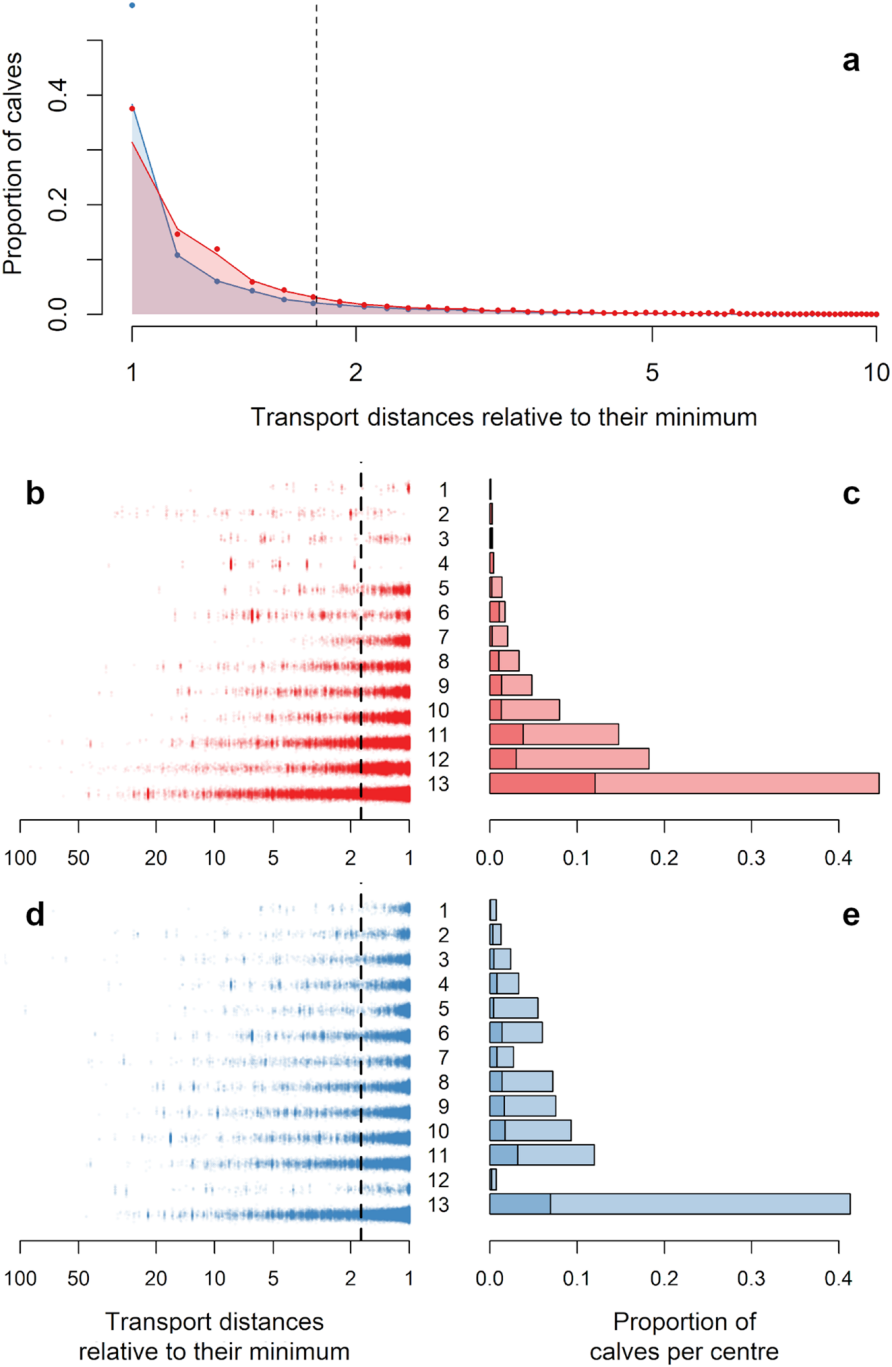
Ratios of historical (red) and optimised (blue) transport distances relative to their minimum (in log scale). (**a**) distribution of the ratios for all the transfers, with a threshold at 1.76 corresponding to the third quartile of the distribution of historical ratios (dashed line). (**b**,**d**) historical (**b**) and optimised (**d**) ratios per centre (numbered in increasing order of calves assigned historically), with the threshold at 1.76 (dashed line). (**c**,**e**) proportion of weaned calves going through each centre, according to the historic (**c**) and optimised assignations (**e**), considering all calves (light colours) or only calves whose ratio is greater than 1.76 (dark colours).

In addition to the absolute transport distances, we also computed the distances relative to the minimum possible distance travelled by a weaned calf, i.e. the transport distance corresponding to a direct transfer from the cow–calf producer to the fattening unit. Thus, we computed ratios of historical and optimised distances over this minimum distance, for each weaned calf. Each ratio indicated the distance each calf travelled in excess compared to an ideal situation. By definition, its value was always greater than or equal to 1, and increased if weaned calves travelled more than the minimum possible distance. The distribution of historical ratios was skewed towards 1, but less than the distribution of optimised ratios (Fig. 2a). We have set a threshold at 1.76 corresponding to the third quartile of the distribution of historical ratios. This means that 25% of the historical transfers were more than 1.76 times the length they would have been if calves had been sent directly from the cow–calf producer to the fattener. Only 19.2% of the optimised ratios exceeded this threshold, and the third quartile of their distributions was 1.39.

The impact of the optimisation was also visible by comparing the historical and optimised ratios separately for each of the 13 centres managed by *Terrena Production Bovine* (Fig. 2b,d). For each centre, the optimised ratios were more closely gathered around one than the historical ratios. The distribution of weaned calves between the centres was also modified by the algorithm (Fig. 2c,e). More than 40% of the transfers still went through the largest centre (numbered 13), but the rest were distributed more evenly among all other centres. In particular, centre 12, with the second-highest number of transfers according to the dataset, was almost never assigned by the algorithm. This change is consistent with each centre’s contributions to the “long-transfer peak” described in the previous section. Indeed, almost all transfers through centre 12 were long transfers (Table 1). By strongly limiting the number of calves sent to centre 12, the algorithm also highlighted its sub-optimal location. It should be noted that the algorithm succeeded in modifying the distribution of calves between the centres while respecting their respective maximum capacities, thus limiting the number of calves in the centres at the same time.

## Discussion

Our algorithm successfully identified potential improvements in the assignment of the weaned calves of the dataset provided by *Terrena Production Bovine* to sorting centre, according to their transport distances. Its impact overwhelmingly visible on long transfers suggest that the transfers most affected were also those with the greatest potential for improvement. This hypothesis is confirmed by looking at the ratios of transport distances relative to their minimum, rather than the absolute values, for which the impact of the algorithm is retained. As its code is freely available, this algorithm could easily be included in a set of decision-support tools used by any cooperative seeking to reduce the transport distances of the livestock it manages. Although a test of the algorithm on a historical dataset is presented here, it can also be used routinely to assign calves to centres. In addition, it could also be used to assess the relevance of the sorting centres for improving transport distances. Indeed, the number of calves assigned to each *Terrena Production Bovine* centre differed substantially from the actual number recorded in the dataset. This result sheds light on the question of the location and maximum capacity of the centres. In particular, the algorithm highlighted a single centre (the 12), whose location was inadequate to limit transport distances, despite being the second-most used overall. This centre was no longer used in the most recent years of the dataset, for reasons independent from the distance travelled. Conversely, several centres were more used by the algorithm, and therefore considered more useful in terms of transport distance, although their maximum capacities were lower. Numerous criteria can guide the choice in the location of sorting centres, including historical, economical, logistical or linked to pre-existing employment pool. Yet, our algorithm provides additional information on transport distance, which could also be taken into account, along with the other criteria, for centre management. For example, running the algorithm without a given centre would provide information on its role in reducing distances travelled by weaned calves. Conversely, running the algorithm with a potential new centre would allow the associated impact on transport distance to be assessed.

The algorithm-induced decrease in the average distance travelled by weaned calves should improve their health and performance. The literature notably establishes a link between transportation and the development of bovine respiratory diseases^8,14,17^ whose incidence is especially important among young bulls during the first weeks of fattening, right after their transfer^15,20^. Moreover, longer transfers are known to be correlated with an increased likelihood of developing symptoms, particularly through their impact on calf stress^21,22^. It should be noted however, that much of the literature on the subject concerns transportation in North America^8,10,14,17^ or Australia^22^, whose associated distances are significantly longer than that considered here (but see Fazio et al.^9^ for a French example of transport distance on stress levels). Unfortunately, information on the quantitative impact of transport distance on cattle welfare and health at European spatial scales is still scarce, and further observational studies would be needed to characterise the impact of our algorithm on welfare and health more precisely. A statistical study on a subset of the dataset presented here showed an overall negative impact of the transport distance on the performance of young bulls during fattening, estimated at − 11 g/day per 120 km travelled over an average fattening period of 313.4 days^13^. These results were obtained on calves managed by *Terrena Production Bovine*, using the same sorting centres and over the same range of transfer distances. On the basis of these results, we could extrapolate an expected gain of 7 g/day of ADG for long distance transfers on average. However, this extrapolation assumes that the impact on ADG increases linearly with distance travelled, so that such a change in distance would have an impact on young bull performance. In addition to the direct impacts on the animals themselves, the optimisation offered by our algorithm could also lead to better profitability for intermediaries such as *Terrena Production Bovine*, whether in terms of fuel efficiency or more efficient management of the occupation of centres. These improvements could be achieved through a more appropriate assignment of calves to centres only and without any other cost.

The functioning of the algorithm we have presented in this study follows a set of constraints. First of all, it had to respect the maximum capacity of each sorting centre. This constraint was respected by (1) processing calves in chronological order and (2) giving priority, among the calves to be processed on the same day, to those whose transport distance would increase the most if their first choice—the centre through which they would have to go to minimize their transport distance—was not available. To assess the impact of these rules, we identified the proportion of calves that were not assigned to their first choice because other calves had priority over them on the same day (see Supplementary Fig. S1). The results showed that 92.6% of calves were assigned to their first choice, and only 1.2% were not assigned to their first or second choice. We also tested removing any capacity limitation by running the algorithm on the dataset from *Terrena Production Bovine* without any maximum capacity (see Supplementary Fig. S2). The results show little impact on transport distances: only 11.2% of the transfers were reduced without maximum capacity and 4.8% of them decrease by more than 25 km. Overall, these results confirm that the way in which the algorithm handled this constraint had only a marginal impact on the distances travelled, and thus on our conclusions.

Secondly, the algorithm relied on a computation of distances as the crow flies rather than the driven distances, as the latter were not available for the transfers listed in the dataset were not available. However, we showed that the road network in the area studied was dense enough so that the distances we computed were reliable approximations (see. Supplementary Figure S3). Indeed, they reliably corresponded to 77.3% of the road distances, which means that the improvement in distances following optimization were expected to be conserved. This correspondence is valid for our study area in France, but it should not be assumed that it is the case everywhere, particularly given the differences between the road networks of various countries. Moreover, computing the actual distances travelled by transport trucks would only make sense if the algorithm reconstructed their exact routes, including the possibility that they visit several farms in a single trip. Optimising such itineraries would be analogous to a traveling salesman problem, which is a NP-complete problem, beyond the scope of this algorithm. Yet, we recommend that cooperatives record these itineraries, as they would provide valuable information, at least to measure actual transfer distances more accurately, and potentially better explain their role in livestock welfare and health.

Thirdly, the algorithm required prior knowledge of the composition of batches, before they arrived in the sorting centres. This would therefore involve handling the design of batches (i.e. choosing which calf to assign) separately from the actual creation of the batches (i.e. grouping the selected calves together before transporting them to fattening units). Obtaining information on most batch design criteria, such as the number, breed or age of calves, should not be a concern, but getting information on bodyweight may be more difficult. Currently, bodyweight homogeneity among the animals is a major criterion and is usually measured on arrival at the centre. Yet, the relevance of this criterion is being questioned^13,23^. Batch composition based on other criteria evaluated before calves are transported is increasingly taken into account by cooperatives such as *Terrena Production Bovine*, and would facilitate the use of the algorithm presented in this study. Even then, sorting centres would remain essential, as the location where animals are grouped in batches before they are sent to a fattener.

Finally, the algorithm only modifies the centre by which a weaned calf goes through. However, it also ensures that the weaned calves only go through a single sorting centre, as it also minimizes the total distance of the transfer. Although this practice tends to be rarer among cooperatives such as *Terrena Production Bovine*, we estimated that at least 8% of the weaned calves of the database went at least through two sorting centres. This proportion necessarily drops to 0 according to the transfers generated by the algorithm. Direct modification of other characteristics of the transfers could improve them further. Firstly, transport distances could be reduced by eliminating transit through a centre altogether when possible. Direct transfer of animals from cow–calf producer to fattener would by definition reduce the distance travelled to a minimum. They represent only 5% of the transfers included in the dataset from *Terrena Production Bovine*, but their share could increase, especially if the design of batches no longer depends on bodyweight measurements at sorting centres. Secondly, an algorithm could also aim to match cow–calf producers and fatteners according to their location. Yet, improvement could be limited by other criteria to be taken into account, such as breed, weight and age. In addition, differences between the areas where cow–calf producers and fatteners are located could result in an irreducible distance that would still have to be travelled by calves, although this may not be the case in all countries or for all industries. However, such improvements are beyond the scope of the algorithm presented here, which aims to improve the distances travelled with the least possible disruption to the logistics of cooperatives.

## Methods

### Presentation of the dataset

**Figure 3:**
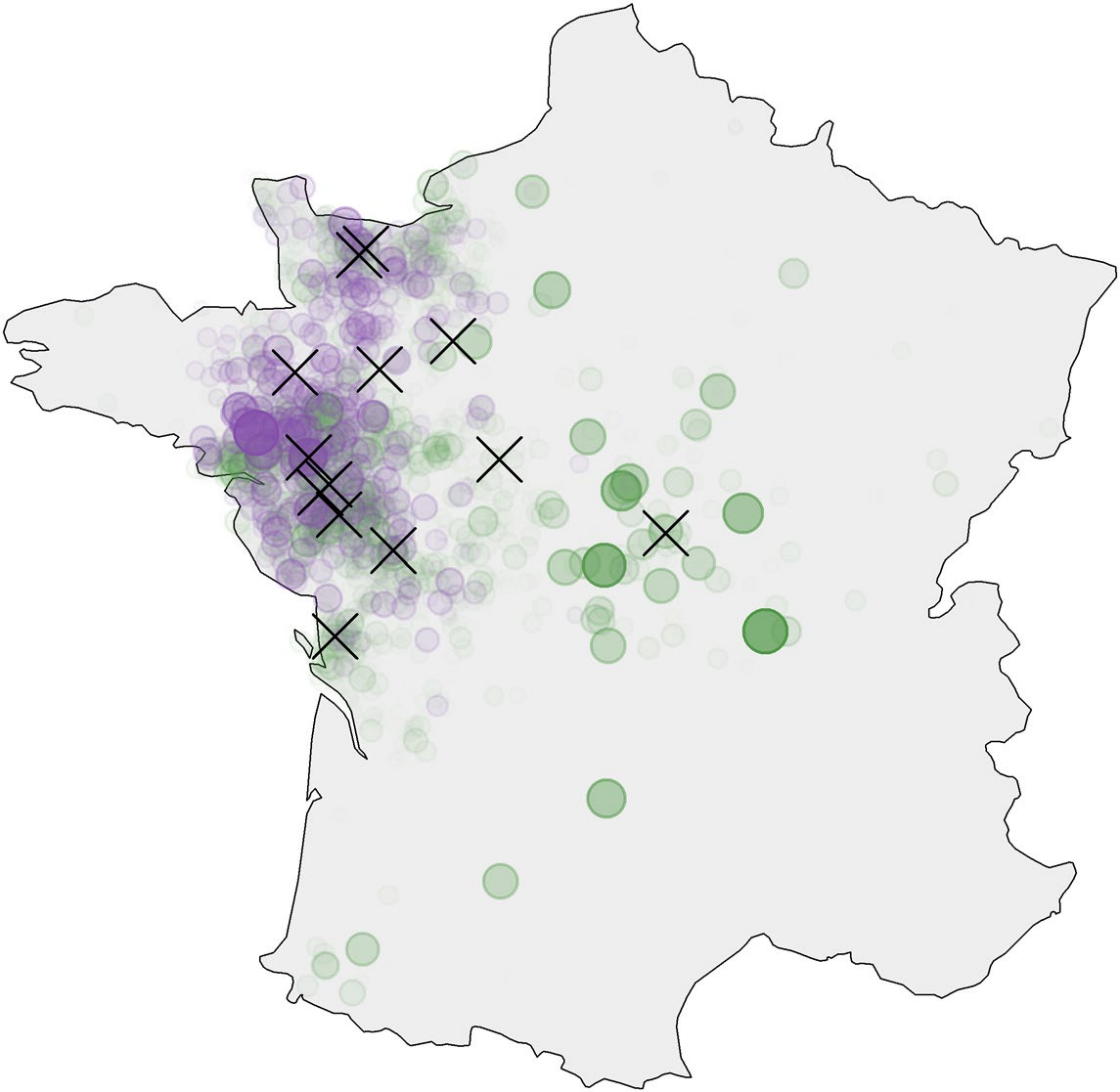
Geographic distribution of the sorting centres (black crosses), calf-cow producers (green circles) and fatteners (purple circles) of the historical database (*Terrena Production Bovine*) in metropolitan France. The size of the circles is proportional to the number of animals coming from or going to each holding.

The dataset presented in this study was provided by *Terrena Production Bovine*, a beef producer organization located in Western France. The data concerns 136,892 weaned calves managed by the organization between 2010 and 2018, and grouped with other calves into one of the 9701 batches sent to fattening farms over the period. The dataset includes the following information: their breed, the cow–calf producer they come from, the batch they were assigned to, the fattener they were sent to, their date of transfer from the cow–calf producer and their date of transfer to the fattener. In total, the dataset lists 3675 different cow–calf producers and 1028 different fatteners. Over the all period, the cooperative operated 13 different centres through which 95% of the weaned calves (129,756 animals in 9383 batches) transited. The first sorting centre each of them was sent to is indicated in the dataset. The sorting centres were numbered for confidentiality reasons, from 1 to 13 by increasing order of number of calves managed. Hence, centre 1 received the least amount of calves of all centres, while centre 13 received the most. The location of the sorting centres is presented in Fig. 3, together with the locations of the cow–calf producers and fatteners. The remaining 5% of the calves (7136 animals in 318 batches) did not go through a sorting centre, but were sent directly from a cow–calf producer to a fattener.

We computed the distances travelled by the weaned calves in the dataset based on the location of each holding. They were all associated with a set of coordinates (latitude, longitude) according to the INSEE code of the commune (a French administrative subdivision) they were in. These coordinates were used to compute distances between two holdings, using the spherical law of cosines. Hence, the distance between holding A and holding B was computed as follows:

$${dist}_{A,B}=R\times {cos}^{-1}\left(sin\left({lat}_{A}\right)\times sin\left({lat}_{B}\right)+cos\left({lat}_{A}\right)\times cos\left({lat}_{B}\right)\times cos\left({lon}_{A}-{lon}_{B}\right)\right)$$

with *lat*_*A*_ and *lat*_*B*_ the respective latitudes of A and B, *lon*_*A*_ and *lon*_*B*_ their respective longitudes, and $R$ the Earth’s radius in km. Hence, we computed distances as the crow flies, which were systematically smaller than the actual driving distances. However, the distances computed corresponded to a consistent proportion of the driving distances in the area covered by the dataset (see Supplementary Fig. S3). Therefore, comparisons between distances computed relative to one another were good approximations of comparisons between the corresponding driving distances.

**Figure 4:**
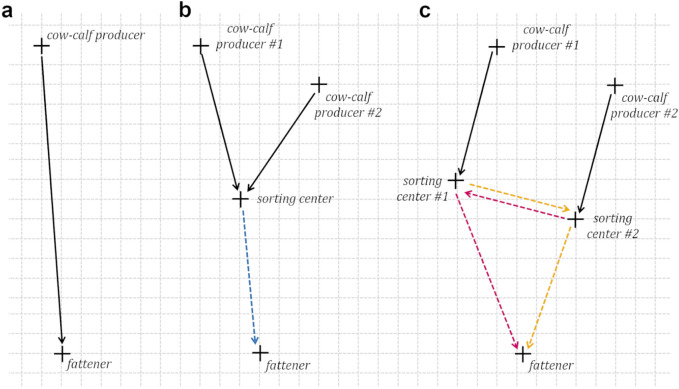
Potential itineraries of the weaned calves of a same batch, including movements provided by the database (solid black arrows) and the inferred movements (dotted arrows). (**a**) The calves not going through any sorting centre move only once. (**b**) When all calves are sent to the same sorting centre, the movement from this centre to the fattener is inferred (blue). (**c**) When calves are sent to two different sorting centres, they can be reunited into the first (purple) or the second (yellow) sorting centre. The centre where they are reunited is the one minimizing the distances travelled (here centre #2).

The transport distance of each calf was computed as the sum of all the distances travelled along its complete itinerary. 5% of them not going through a sorting centre moved only once: from the cow–calf producer to the fattener (Fig. 4a). The remaining 95% moved at least twice: from the cow–calf producer to their first sorting centre, and from their last sorting centre to the fattener. In between, the calves potentially also moved between centres. However, the dataset only informed us about the first sorting centre each weaned calves went through, but not about their complete itinerary. To circumvent this lack of information from the database, we chose to infer the rest of the itinerary with the following rule of thumb: the distances travelled had to be as short as possible. We therefore considered the best case scenario distance-wise for the historical data. While the actual itineraries were likely longer than what is assumed, this method ensures that any improvement brought about by our algorithm can be viewed as a lower bound for the actual improvement When all the weaned calves of a same batch were initially sent to the same sorting centre (i.e. 84% of the batches of the dataset), we assumed that it was the only one they went through. Therefore, they moved twice: from the cow–calf producer to the centre and from the centre to the fattener (Fig. 4b). When the weaned calves of a same batch were initially sent to multiple sorting centres (i.e. 13% of the batches), we assumed that they were only moved one more time to be reunited in one of these sorting centres before being sent to the fattener. The centre where they were reunited was chosen so that the sum of the total distance travelled by all the calves in the batch, from their cow–calf producer to their fattener, was minimum. Therefore, those initially sent to the centre where they were reunited were considered to move twice, and those initially sent to other centres moved three times: from the cow–calf producer to their original centre, from this centre to another one, and from this last centre to the fattener (Fig. 4c). Overall, of the 95% of calves not directly sent to their fattener, 87% went through a single sorting centre, and 8% went through two centres.

### Presentation of the algorithm

The algorithm we developed aimed at choosing to which sorting centre the weaned calves should be sent in order to minimise their transport distance. It used two kinds of inputs: a list of the calves to be transported and a list of the available sorting centres. It returned a single output: a list of one and only one centre for each calf. It should be noted that the algorithm only selected centres, but did not alter the batch compositions or the fattener they were sent to. Therefore, the algorithm did not handle transfers from the cow–calf producer directly to the fattener, as these animals already did not go through any centre. The list of calves used as input by the algorithm included the following information: their ID, the locations of their cow–calf producer and of their fattener, the batch they belong to, their dates of departure from their cow–calf producer and their date of arrival at their fattener. The list of sorting centres used was provided with the following information: their maximal capacity, i.e. the largest number of calves that could be simultaneously in a given centre. The history of the maximum capacities of the centres managed by *Terrena Production Bovine* was not available for this study. Therefore, it was estimated for each centre and each year, as the maximum number of weaned calves present in the centre during the year. These values therefore corresponded to the lowest possible estimate of the maximum capacity of each centre.

**Figure 5:**
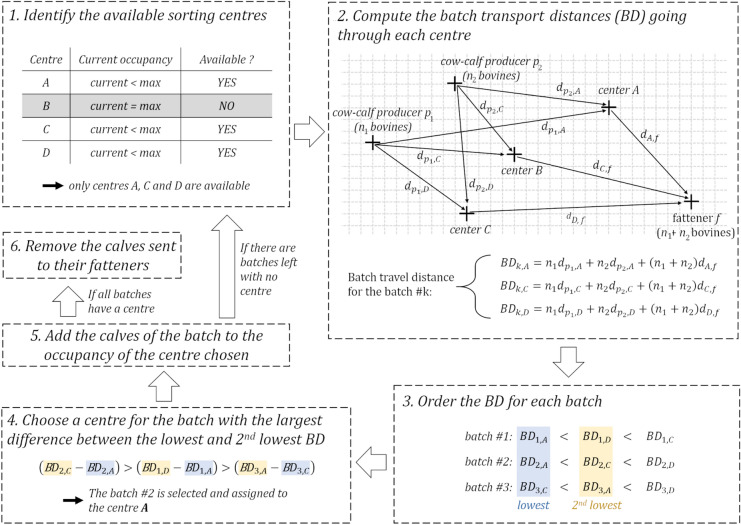
Schematic representation of the algorithm for a given date. The algorithm identifies centres that are already at their maximum capacity (step 1, in grey) and computes the batch transport distance (BD) for all the batches, if they went through each available centre (step 2). It identifies the lowest and second lowest values of BD for each batch (step 3) and selects the batch for which the difference is the largest (step 4). The selected batch is assigned to the centre with the lowest BD (centre A in the example) and the calves are added to the occupancy of the centre (step 5). The first five steps are repeated until all the batches are assigned to a centre. Then, the calves sent to their fatteners on the given date are removed.

The algorithm managed calves by batches, in the chronological order of their date of departure from their cow–calf producer. For a given date *t*, the algorithm functioned as follows (Fig. 5):

1. The algorithm assigned a current occupancy to each centre, corresponding to the number of calves already present. A centre was considered available only if its current occupancy was lower than its maximal capacity.
2. The algorithm computed the batch transport distance of every batch *b* considered at time *t*, going through each available sorting centre *s*, noted *BD*_*b,s*_. This distance was equal to the sum of the distances travelled by every calf of batch *b,* if they went through centre *s*, and is computed as follows:
  $$B{D}_{b,s}=\sum_{c}\left({n}_{c}{d}_{{p}_{c}s}\right)+\sum_{c}\left({n}_{c}\right){d}_{f,s}$$
  with *n*_*c*_ the number of calves coming from cow–calf producer *c*, *d*_*pc,s*_ the distance between cow–calf producer *c* and sorting centre *s*, and *d*_*f,s*_ the distance between the fattener *f* and the sorting centre *s*.
3. For each batch, the algorithm ranked the different values of *BD*_*b,s*_ (one for each available sorting centre) in increasing order.
4. The algorithm identified the batch for which the difference between the lowest value of *BD*_*b,s*_ and the second-lowest value of *BD*_*b,s*_ was the largest. The centre selected for the calves in the batch was the one associated with the lowest value of *BD*_*b,s*_.
5. The current occupancy of the centre selected was increased by the number of calves assigned.
6. If there were batches left to assign, the algorithm restarted at 1. Otherwise, it removed the calves sent to their fatteners at time *t* from their respective centres and went on to *t* + *1*.

## Supplementary information

Supplementary Information.

## Data Availability

The code of the algorithm, as well as the script used to compute the indirect and direct distances from historical datasets, the script used to produce the figures presented in the manuscript and a test dataset are freely available at: https://sourcesup.renater.fr/projects/pub-dist-algo.
