# Supplementary material for: Selecting sorting centres to avoid long distance transport of weaned beef calves
Source: Sci Rep. 2021 Jan 14;11:1289. doi: 10.1038/s41598-020-79844-4 (PMC7809358; doi:10.1038/s41598-020-79844-4)
Supplement: Supplementary file 1 — Supplementary Information. [file 41598_2020_79844_MOESM1_ESM.pdf]

Supplementary information for the article *Selecting sorting centres to avoid long distance transport of weaned beef calves*

Authors: T. Morel-Journel[1], E. Vergu[2], J.-B. Mercier[3], N. Bareille[1], P. Ezanno[1]

[1] INRAE, Oniris, BIOEPAR, 44300, Nantes, France
[2] INRAE, Université Paris-Saclay, MaIAGE, 78350, Jouy-en-Josas, France
[3] Terrena Innovation, La Noëlle, 44155 Ancenis, France

This supplementary information includes:

- Supporting Figure S1: *Impact of the priority order used for the algorithm*
- Supporting Figure S2: *Impact of the centre maximal capacity*
- Supporting Figures S3 & S4: *Comparison between driving distances and computed distances with the spherical cosine law*

Impact of the priority order used for the algorithm

We assessed the impact of the priority order used to assign batches handled the same day to their sorting centre. In the algorithm the batches are handled by decreasing order of difference between the lowest and second lowest batch distances (i.e. the sum of the distances travelled by all the weaned calves of a same batch). Thus, centres which would increase the most their travelled distance by not being assigned to the available centre minimizing their batch distance are handled first.

At each date, we ranked the *n* available centres from 1 to *n*, in increasing order of batch distances, for each batch. For each assignment, we recorded the rank of the centre assign to each batch. Hence, we recorded 1 if the batch was assigned the first centre (i.e. the centre associated with the lowest batch distance), 2 if it was assigned to the second centre (i.e. the centre associated with the second lowest batch distance).

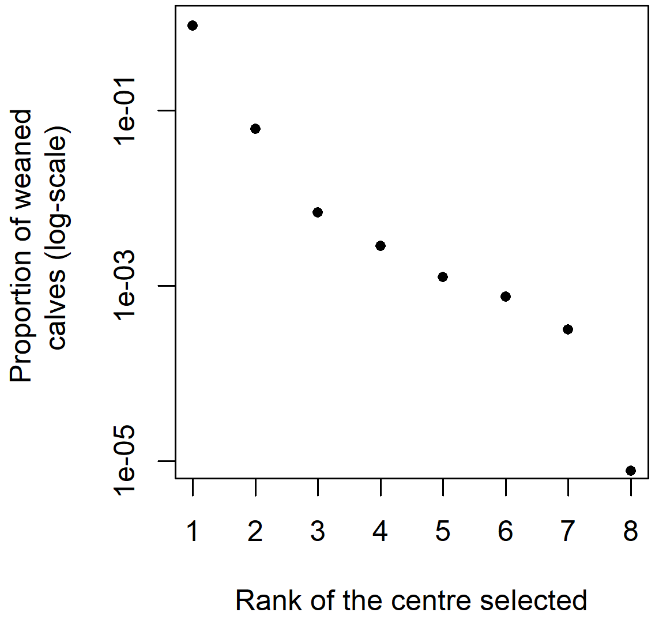

Figure S1: Distribution of the ranks of the centres selected for each weaned calves by the algorithm. The y-axis (proportion of weaned calves assigned their xth preferred centre) is on a log scale because the overwhelming majority of calves (92.6%) were assigned the best centre.

Results show that 92.6% of the weaned calves were actually assigned to the centre available on this date minimizing the batch distance. 6.2% were assigned to the second best centre, 0.7% to the third best centre and 0.5% to a centre worse than the third best. Therefore, respecting the priority ordered imposed by the algorithm only affected 7.4% of the weaned calves, and only 1.2% were not assigned to the best or second best centre available this day.

Impact of the centre maximal capacity

To assess the impact of taking any centre maximal capacity at all on the results, we performed another assignment where the availability of the centres was ignored. Hence, all the weaned calves were assigned to the centre minimizing the batch distance (i.e. the sum of the distances travelled by all the weaned calves of a same batch), without any restriction. We then compared the distribution of indirect distances according to this assignment with the original assignment performed in the study in two ways: overall, and more specifically for the weaned calves for which the distance travelled changed.

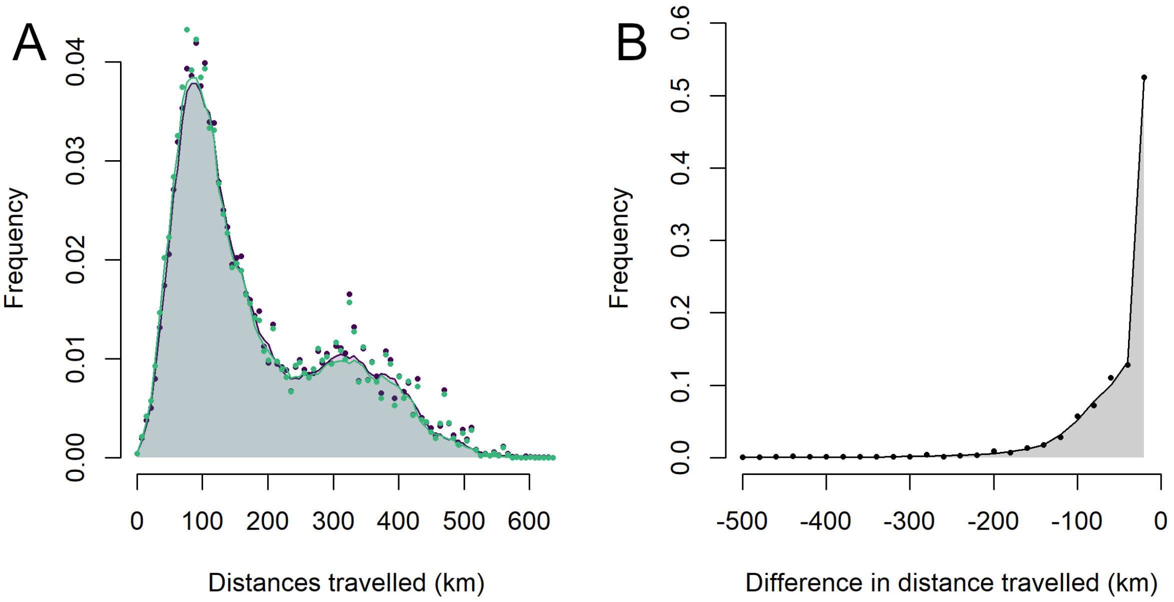

Figure S2: Comparison of the distance travelled by the weaned calves with and without maximal capacities for the centres. **A:** Distribution of the distances travelled by each weaned calf after optimization of the algorithm with (purple) and without (cyan) maximal capacities for the centres. **B:** distribution of the decrease in the distance travelled by the weaned when removing the maximal capacities for the centres, for the calves with a decreased distance travelled.

The assignment without any maximal capacity affected the travel distances of 14,604 calves (11.2%). These changes resulted in a slight shift of the overall distance distribution towards lower values (Figure s2a): the average distance travelled is reduced from 189km to 184km, the low-distance peak is slightly increased and the high-distance peak is slightly decreased). When looking at the changes in distance travelled by the weaned calves, 8,293 of them (56,8% of them) showed a difference lower than 25km between the assignment with and without maximal capacities. Only 1,569 (10,7% of the calves whose travel distance was affected, 1.2% of the total) showed a difference greater than 100km.

Comparison between driving distances and computed distances with the spherical cosine law

We considered the distances between two set of locations (i) the 13 sorting centres considered for the study and (ii) a grid of locations in the area where the holdings considered for the studies are located. This grid spans from 46 to 48.4 degrees North and from 2 degrees West to 1.2 degrees East, with a location every 0.4 degree of latitude and of longitude (Figure S3a). The locations not on land are ignored, for a total of 60 locations. Hence, we considered 13 x 60 = 780 distances. These distances were computed in two ways:

- By using the method used in the study, i.e. using the spherical law of cosines
- By recording the driving distance between the two locations according to *Google Maps* (as recorded the 15/05/2020).

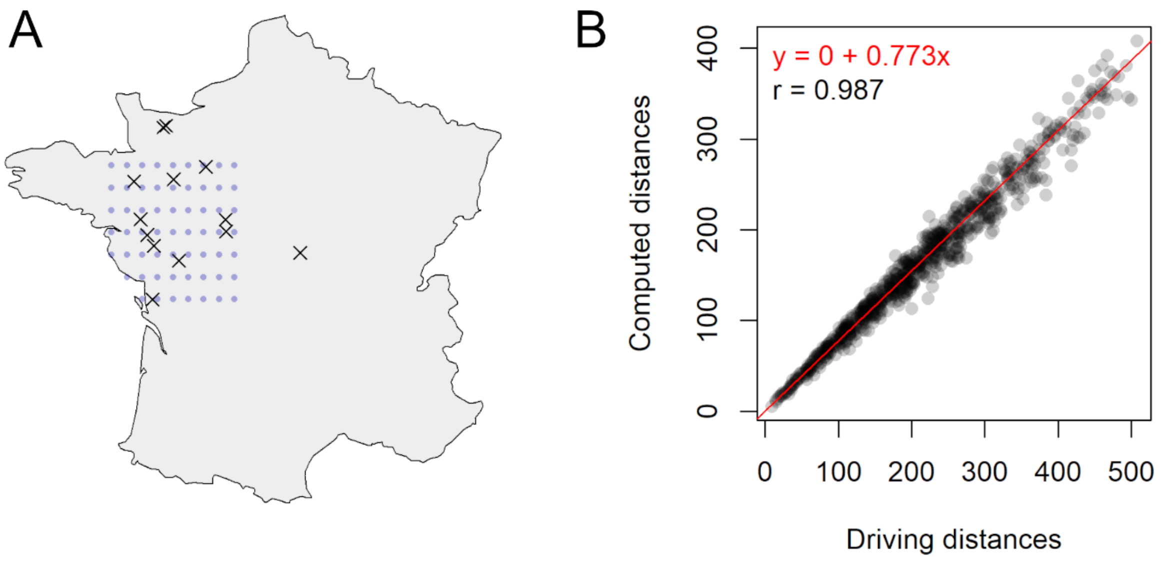

Figure S3: Comparison between driving distances and computed distances with the spherical cosine law. **A:** Locations of the sorting centres considered for the study (black crosses) and locations on a grid in the area where the holdings considered are located (blue dots). **B:** Distances between the centres and the locations on the grid as the crow flies as a function of the driving distances (black dots). The red line corresponds to the equation of the average ratio between the computed and the driving distance, i.e. the computed distances are 77.3% of the driving distances on average.

The results show that the computed distances are strongly positively correlated with the driving distances (Pearson's r = 0.987, $p < 1e^{-4}$). The computed distance consistently corresponds to 77.3% of the driving distances (with 95% of the ratios of computed to driving distances between 64.5% and 87.9%). Hence, the distances computed as the crow flies in the study can be considered a reliable estimation of the actual driving distance for the dataset considered.

These driving distances were recorded assuming that trucks transporting cattle could use toll roads. In order to assess if this assumption could have an impact on the road distance, we compared distances with and without toll roads in the area studied. We randomly drawn 200 couples of locations in the area considered above, separated with a driving distance between 20km and 450km. For each couple, we computed road distance between each point using *Google Maps*, with and without the options "Avoid tolls" and "Avoid highways" (Figure S4).

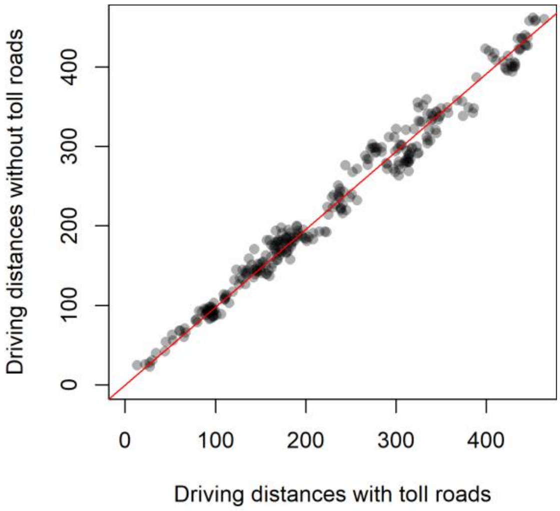

Figure S4: Relationship between driving distances including itineraries using toll roads and those not including them (black dots). The red line corresponds to the equation of the ratio between the two, which is of 97.7%.

The results show that the driving distances using toll roads or not using toll roads are also very strongly correlated (Pearson's r = 0.991, $p < 1e^{-4}$), and very close to one another. This shows that the use of highways or secondary roads has little impact on the distance driven for movements, for the area considered.
